# The role of topoisomerase I in suppressing genome instability associated with a highly transcribed guanine-rich sequence is not restricted to preventing RNA:DNA hybrid accumulation

**DOI:** 10.1093/nar/gkv1152

**Published:** 2015-11-02

**Authors:** Puja Yadav, Norah Owiti, Nayun Kim

**Affiliations:** Department of Microbiology and Molecular Genetics, University of Texas Health Science Center at Houston, Houston, TX 77030, USA

## Abstract

Highly transcribed guanine-run containing sequences, in *Saccharomyces cerevisiae*, become unstable when topoisomerase I (Top1) is disrupted. Topological changes, such as the formation of extended RNA:DNA hybrids or R-loops or non-canonical DNA structures including G-quadruplexes has been proposed as the major underlying cause of the transcription-linked genome instability. Here, we report that R-loop accumulation at a guanine-rich sequence, which is capable of assembling into the four-stranded G4 DNA structure, is dependent on the level and the orientation of transcription. In the absence of Top1 or RNase Hs, R-loops accumulated to substantially higher extent when guanine-runs were located on the non-transcribed strand. This coincides with the orientation where higher genome instability was observed. However, we further report that there are significant differences between the disruption of RNase Hs and Top1 in regards to the orientation-specific elevation in genome instability at the guanine-rich sequence. Additionally, genome instability in Top1-deficient yeasts is not completely suppressed by removal of negative supercoils and further aggravated by expression of mutant Top1. Together, our data provide a strong support for a function of Top1 in suppressing genome instability at the guanine-run containing sequence that goes beyond preventing the transcription-associated RNA:DNA hybrid formation.

## INTRODUCTION

Genome instability—recombination and mutagenesis—is elevated by highly active transcription (1–7) (see (8,9) for review). The transcription complex itself can physically obstruct other DNA metabolic processes such as replication, especially when the direction of the transcription is in a collisional orientation with the movement of replication fork (10–12). The unwinding of the duplex DNA, mandatory for transcription process, transiently produces single stranded DNA regions of the non-transcribed strand (NTS) that are highly susceptible to chemical and enzymatic modifications. This effect can be aggravated when the nascent RNA stably anneals to the template DNA strand (transcribed strand – TS) producing extensive stretches of RNA:DNA hybrids or R-loops (13). When unresolved, R-loops lead to genome instability additionally by obstructing DNA replication (14) or by generating DSB *via* structure specific endonucleotic activity (15). R-loop formation in lieu of duplex DNA restoration ensues when transcription elongation or important co-transcriptional processes such as splicing, ribonucleoprotein (RNP) assembly and resolution of DNA topological stress are disturbed (16–18). Genome instability associated with R-loops is suppressed by RNase H enzymes, which degrades RNA hybridized to DNA and thereby prevents nucleation of RNA:DNA hybrids (19–21). Extensive R-loop accumulation, particularly at the highly transcribed rDNA locus and the tRNA genes, is observed in absence of this family of enzymes (22,23).

Conditions conducive to R-loop formation—open chromatin structure, DNA strand separation, and negative helical torsion—are similarly conducive to the structural transition of the duplex B-form DNA into non-canonical secondary structures when the transcribed regions contain repetitive sequences (24,25). Genome instability associated with these secondary structure-forming sequences becomes exacerbated under high transcription conditions, which promotes the folding of repetitive DNA strands into the non-B structure formation on the NTS as well as the stable formation of RNA:DNA hybrids involving the TS (26,27).

The four-stranded G-quadruplexes or G4 DNA, which contains a stacked array of multiple G-quartets comprised of four guanines interacting in a planar configuration, is another non-B secondary DNA structure capable of inducing genome instability by obstructing normal DNA transactions (28–31). Sequence motifs with potential to form G4 DNA are enriched at telomeres and G-rich immunoglobulin switch regions and frequently associated with unstable genomic loci including proto-oncogenes (32). Recent bioinformatic analyses of genes involved in chromosomal translocations in lymphoid cancers identified potential G4 DNA-forming sequences near many of the mapped translocation breakpoints (33). As for the immunoglobulin switch regions, chromosomal translocations involving this locus have long been observed in various cancer types (34). For several of the translocation-linked G4 motifs Including those at the major breakpoint region of the *BCL2* gene involved in *t*(14;18) translocation in follicular lymphoma (35) and the *TCF3* gene involved in *t*(1;19) in acute lymphoblastic leukemia (36), the transformation into the quadruplex structure have been confirmed *in vitro*. In the model organism *Saccharomyces cerevisiae*, the capacity of G4 DNA-forming sequences to stimulate repeat instability and gross chromosomal rearrangements has been demonstrated using reporter assays (37–40).

During transcription, the negative and positive topological stress accruing behind and in front of the RNA polymerase complexes, respectively, are removed by topoisomerase I or Top1 (41). Yeast Top1, a type IB topoisomerase, binds DNA non-specifically and generates a transient nick in DNA by covalently attaching to the 3′ end of cleaved strand through the catalytic tyrosine. After swiveling of the DNA strands, the nicked DNA is re-ligated by the 5′ hydroxyl group on the other side of the strand break attacking the phospho-tyrosyl bond thereby releasing Top1 from the DNA end. Top1 protein mutated at the highly conserved active site tyrosine (i.e. yeast Top1Y727F) cannot cleave DNA but forms a non-covalent complex with DNA (42). Interfering with the re-ligation activity of Top1, either by mutation of another conserved tyrosine (i.e. yeast Top1Y722A) or by the Top1-binding camptothecin-class of drugs, results in accumulation of unresolved covalent complex composed of Top1 and nicked DNA (Top1cc) that is cytotoxic and recombinogenic (43).

We previously reported that, in absence of Top1, a guanine-run (G-run) containing mouse immunoglobulin (Ig) switch Mu region sequence (Sμ) integrated into the yeast genome induces high rates of gene conversions, mitotic crossovers and gross chromosomal rearrangements including loss of chromosome arm and large segmental duplications (44,45). The genome instability was suppressed when the level of transcription was reduced and when the direction of transcription was altered to place the guanine-runs on the TS. In absence of Top1 activity, negative torsional stress increases, especially at highly transcribed regions. The re-annealing of TS to the NTS becomes less efficient in this condition, thereby shifting the equilibrium to hybridizing with the nascent RNA. At the Sμ sequence containing multiple runs of guanines, defective Top1 function also facilitates formation of G4 DNA structures by producing both negatively supercoiled NTS and single-stranded regions on the NTS necessitated by RNA:DNA hybrids. We initially hypothesized that the R-loop accumulation is the major underlying cause of the G4-associated genome instability associated with Top1-deficiency. In this report, we present a clear *in vivo* demonstration of sequence-specificity in R-loop accumulation; when the G4-forming Sμ is highly transcribed, RNA:DNA hybrids accumulate in an orientation-specific manner that correlates with the orientation-specific increase in genome instability observed in Top1- or RNase H-deficient genetic backgrounds. In absence of Top1, however, RNA:DNA hybrid accumulation did not fully account for the elevated recombination at the G-run containing sequence. Our data further show that the removal of negative helical stress during transcription is necessary but not sufficient for the Top1-mediated suppression of genome instability induced by the G-runs.

## MATERIALS AND METHODS

### Yeast strains and plasmids

All yeast strains used here were derived from YPH45 (*MATa, ura3-52 ade2-101 trp1Δ1*; (46)). The construction of *pTET-LYS2* and *pTET-lys2-GTOP* or -*GBTM* (previously referred to as *pTET-lys2-SμF* and *-SμR*) cassettes and the genomic integration on chromosome were previously described (7,44). Gene deletion was carried out through one-step allele replacement by amplification of the loxP-flanked marker cassettes (Euroscarf; (47)). Two-step allele replacement procedure was used to introduce the *pol2M644L* mutation as previously described (48).

*CEN* plasmids expressing *WT TOP1, top1 Y727F* and *top1 Y722A* contain endogenous *TOP1* promoter and *URA3* marker. pSTS77 (49) and YEptopA-PGAL1 (50) are 2μ plasmids containing the yeast *GAL1* promoter upstream of the fusion protein of *E. coli* gyrB and gyrA (ecGyrase) or *E. coli* Top1 (ecTop1), respectively, and were gifts from Dr Joaquim Roca (Molecular Biology Institute of Barcelona, Barcelona, Spain). For vector controls, a CEN plasmid pRS416 or a high copy 2μ plasmid YEP24 was used as indicated.

### Determination of recombination rates

Gene conversion rates were determined according to the growth and plating conditions as previously described (44). For complementation assays, yeast strains were transformed with the appropriate plasmid DNA and plated on synthetic complete media with 2% glucose lacking uracil (SCD-Ura) plates. Individual Ura^+^ transformants were used to inoculate 1 ml cultures in SC-Ura media supplemented either with 2% glucose (for pRS416, *WT TOP1, top1 Y727F* and *top1 Y722A*) or with 1% raffinose plus 2% galacotse (YEP24, ecGyrase and ecTop1). After 4 day's growth at 30°C, appropriate dilutions of the cultures were plated on SCD-Ura for determination of total cell numbers or on SCD-Ura-Lys for determination of recombinants. For each strain, 12–36 cultures were used to determine rates and 95% confidence intervals using the Lea-Coulson method of median (51,52).

### Survival and recombination frequency in CPT-treated cells

Overnight cultures in YEPD (1% yeast extract, 2% peptone, 2% glucose) were diluted to 2 × 10^5^ cell/ml in fresh YEPD. Camptothecin (CPT, Sigma) to the final concentration of 100 μM or dimethyl sulfoxide (DMSO) was added to each 2 ml cultures and cultured for 24 h at 30°C on a roller drum. The cells were harvested and diluted appropriately before plating on YEPD plates to determine the total cell number and on SD-Lys plates to determine the number of recombinants. 8–24 cultures were used to determine the recombination frequency for each strain.

### RNA:DNA hybrid immunoprecipitation

Yeast genomic DNA was prepared from fresh 5 ml cultures grown in YEPD by agarose plug methods by first suspending the cells at the density of 100 mg cells/1ml of 1% low melting agarose. For each immunoprecipitation (IP), DNA in three agarose plugs were dissolved in GenElute Gel extraction solution (Sigma Cat# NA1111) and fragmented to average size of 750 bp using the QSonica sonicator equipped with a microtip. DNA fragments were extracted according to the GenElute Gel extraction kit protocol and resuspended in 700 μl of IP wash solution (0.1% deoxycholic acid, 1 mM EDTA, 50 mM HEPES pH7.5, 140 mM NaCl, 1% Triton X-100). For each IP, 10 μl of protein A-conjugated dynabeads (Life Technologies) were incubated with 2.5 μg of S9.6 antibody (a gift of Dr. Stephen Leppla, NIH/NIAID), washed with IP wash solution and added to the DNA preparation. After incubation at 4°C for 16–20 h, the beads are washed three times with the IP wash solution and once with TE. Both IP and input samples were suspended in 80μl of elution buffer (50 mM Tris–HCl pH7.5, 10 mM EDTA, 1% SDS) and 20 μl of pronase (20 mg/ml, Roche) and incubated for 2 h at 42°C followed by 8 h at 65°C. DNA was purified using the Qiagen MiniElute PCR Purification Kit prior to qPCR using SensiFAST SYBR no-ROX Mastermix (Bioline) and CFX Connect instrument (Biorad). Cycling conditions were 95°C for 3 min followed by 40 cycles of 95°C for 5 s, 60°C for 10 s, and 72°C for 10 s. Primers used in the qPCR analysis are listed in the Supplementary Table S1.

## RESULTS

### Top1-mediated suppression of genome instability at the highly transcribed G-run sequence requires its catalytic activity

**Figure 1.**
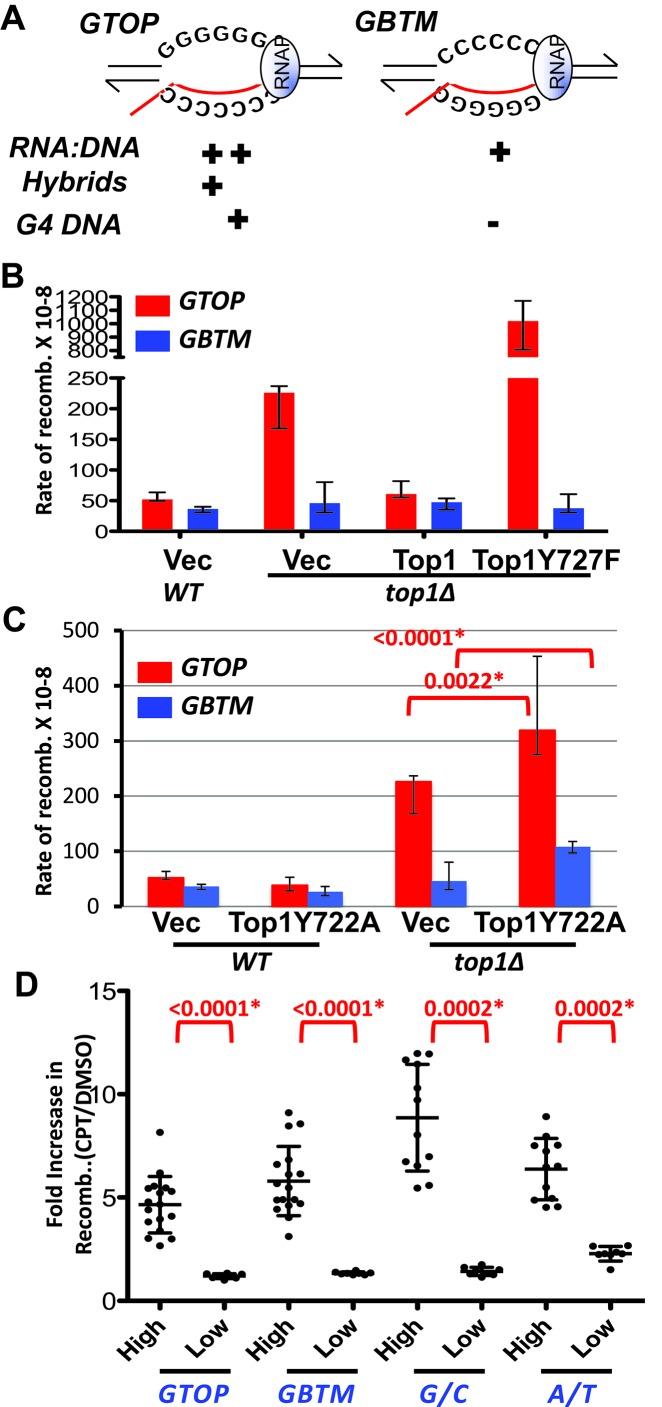
Effects of WT or mutant Top1 on the rates of recombination at the *pTET-lys2-GTOP* and *-GBTM* reporters. (**A**) Transcription orientations and the guanine-runs in the Sμ-containing cassettes. Guanine-runs are located on the non-transcribed or the transcribed strand in the *pTET-lys2-GTOP* or *pTET-lys2-GBTM* cassette, respectively. The red line indicates the nascent RNA. The sequence of Sμ in *-GTOP* orientation is listed in Supplementary Figure S1A. Relative abundances of RNA:DNA hybrid are inferred from Belotserkovskii *et al*. (54). For B, C, and D, the rates of recombination at the *pTET-lys2-GTOP* (red bar) or *pTET-lys2-GBTM* (blue bar) cassette. The rates were determined by the method of median. Error bars indicate 95% confidence interval. (**B**) *WT* or *top1Δ* cells transformed with the indicated plasmid were grown in synthetic media lacking uracil supplemented with 2% glucose. Vec: pRS416. (**C**) Recombination induced by Top1Y722A. *WT* or *top1Δ* cells transformed with the indicated plasmid were grown in synthetic media lacking uracil supplemented with 2% glucose. Vec: pRS416. The rates of recombination at the *pTET-lys2-GTOP* (red bar) or *pTET-lys2-GBTM* (blue bar) cassette. The rates were determined by the method of median. Error bars indicate 95% confidence interval. P values (shown above red brackets) were determined by Mann-Whitney test using the Prism software. *, significantly different; ns, no significant difference. (**D**) Recombination induced by CPT treatment. WT cells containing the indicated *pTET-lys2-*derived reporter construct were treated either with DMSO or CPT (see material and methods). Each dot indicates the frequency of Lys+ colonies in CPT treated cells divided by the frequency in DMSO treated cells. Cells were cultured in YEPD (‘High’ transcription condition) or YEPD + 2 μg/ml doxycycline (‘Low’ transcription condition) as indicated in the x-axis label. Error bars indicate standard deviation from 8 to 24 independent measurements. *P values were determined by Mann–Whitney test using the Prism software.

We previously reported on the construction of recombination reporter assay that allowed us to determine the contribution of guanine-run containing sequences to genome instability under high- or low-transcription conditions (44). In this reporter assay, a 770-bp fragment of murine immunoglobulin Switch Mu sequence (Sμ) was inserted into the *LYS2* gene in the yeast genome. The Sμ fragment (Pubmed Accession #J00442), comprising 46% guanines overall and 35 runs of three or more guanines, was inserted either in its physiological or ‘*GTOP*’ orientation with the top or non-transcribed strand (NTS) containing the G-runs or in inverse or ‘*GBTM*’ orientation with the bottom or the transcribed strand (TS) containing the G-runs (Figure 1A and Supplementary Figure S1). This reporter construct (*pTET-lys2-GTOP* or -*GBTM*) replaced the *HIS4* gene located on the left arm of Chromosome III and is regulated at the transcription level by the tetracycline-repressible promoter. Based on the demonstrated thermodynamic stability of rG:dC basepairs (53) and the *in vitro* analysis implicating more stable RNA:DNA hybrid formation when the RNA strand contains high guanine content (54), we postulated that the *pTET-lys2-GTOP* construct has higher potential for R-loop accumulation than the *pTET-lys2-GBTM* (+++ versus + in Figure 1A). And G4 DNA assembly is much more likely to occur at the *pTET-lys2-GTOP* construct, where the guanines in the NTS become unpaired and are free to form the Hoogsten bonding with other guanines during transcription (+ versus – in Figure 1A).

In absence of Top1, gene conversion, mitotic crossover and gross chromosomal rearrangements at the guanine-run containing Sμ fragment are significantly elevated in transcription- and orientation-dependent manner (44,45). In order to determine whether the catalytic activity of Top1 is required for the suppression of genome instability, we transformed the *top1Δ* yeast strains containing either *pTET-lys2-GTOP* or *pTET-lys2-GBTM* construct with plasmids carrying WT *TOP1* gene or the *top1Y727F* allele and measured the rates of gene conversion occurring at these reporter constructs. With the catalytic tyrosine (Y727) mutated to phenylalanine, the Top1Y727F mutant cannot form the phosphotyrosyl bond necessary to cleave DNA but retains DNA binding activity (55–57). The recombination rate of the *pTET-lys2-GBTM* reporter was not significantly changed when WT Top1 or Top1Y727F was expressed (Figure 1B). For the *pTET-lys2-GTOP*, the gene conversion rate was decreased by ∼4-fold with WT Top1 expression and became indistinguishable compared to the rate in WT cells. With Top1Y727F expression, the recombination rate for the *pTET-lys2-GTOP* was 17-fold higher compared to WT Top1 expression and 4.5-fold higher compared to vector control. When the transcription of the *pTET* promoter was repressed by the addition of 2μg/ml doxycycline in the growth media, Top1Y727F expression did not significantly elevate recombination rates for either the *pTET-lys2-GTOP* or *pTET-lys2-GBTM* construct indicating the hyper-recombinogenic effect of Top1Y727F is transcription-dependent (Supplementary Figure S2A).

We reported previously that the recombination rate in *WT* strains for the *pTET-lys2-GTOP* construct is higher than the *pTET-lys2-GBTM* by about 2-fold (44). This relatively small difference in the rates of recombination was nevertheless statistically significant. To test whether the increased level of Top1 activity can overcome the elevated recombination associated with the *pTET-lys2-GTOP* construct, we transformed a WT Top1-expressing plasmid into the WT strains containing the *pTET-lys2-GTOP* or *pTET-lys2-GBTM* construct. With the control vector, the recombination rate for the *pTET-lys2-GTOP* is 1.4 fold higher than the rate for the *pTET-lys2-GBTM* and this difference is statistically significant (*P* = 0.0009) (Supplementary Figure S2B). Upon WT Top1 expression from the plasmid, the rate of recombination for the *pTET-lys2-GTOP* was reduced to a level that is statistically indistinguishable from the *pTET-lys2–GBTM* (*P* = 0.141) indicating that the limiting factor leading to the relatively higher recombination rate for the *pTET-lys2-GTOP* construct in WT strains is Top1 activity. In WT backgrounds, Top1 Y727F expression did not significantly change the recombination rate for the *pTET-lys2-GTOP* or *pTET-lys2-GBTM* construct.

### Recombination induced by the Top1-DNA complex is dependent on the level of transcription

Yeast Top1 with mutation of tyrosine to alanine at the amino acid 722 (Top1Y722A) has intact DNA cleavage activity but significantly reduced re-ligation activity leaving Top1 trapped as a Top1–DNA cleavage complex (Top1cc) (43). Accumulation of Top1cc due to Top1Y722A mutant was shown to elevate recombination between homologous chromosomes in diploid yeast cells and instability at the rDNA and *CUP1* tandem arrays (58) and to enhance the 2-nucleotide deletion mutations occurring at short tandem repeat sequences (59). In order to determine whether the genome instability induced by Top1cc is enhanced by G4 DNA structures, we expressed Top1Y722A from a plasmid with the endogenous *TOP1* promoter and measured the recombination rate at the highly expressed *pTET-lys2-GTOP* or -*GBTM* reporter. In WT strains with functional genomic allele of *TOP1*, Top1Y722A expression from the plasmid did not have significant effect on the rates of recombination (Figure 1C). When Top1Y722A was expressed in *top1Δ* backgrounds, the recombination rates for both the *pTET-lys2-GTOP* and -*GBTM* were significantly elevated.

Top1 is the target of a widely used anti-cancer chemotherapeutic named campthothecin (CPT) and its derivatives, which bind to Top1-DNA covalent complex and inhibits the re-ligation step (41,60). Similar to Top1Y722A expression, Top1cc accumulate in the genome when treated with CPT. We tested whether CPT treatment can elevate recombination at the Sμ-containing reporter constructs. Recombination frequencies in cells treated with CPT were 5- and 6-fold higher than DMSO treated control cells for the *pTET-lys2-GTOP* and -*GBTM*, respectively (Figure 1D). In addition to the 16 GGGG•CCCC runs, Sμ fragment inserted into the *LYS2* gene for the construction of the *pTET-lys2-GTOP* and *-GBTM* reporter is 60% G/C rich (Supplementary Figure S1A). We tested whether the GC content affects the sensitivity to CPT by measuring CPT-induced recombination at the identical *pTET-lys2* reporter with insertion of a 750 bp fragment of chicken β-globin 2 coding sequence. This fragment is 63% GC and contains only four widely spaced G-runs (‘G/C’ in Figure 1D). We also tested the effect of CPT treatment on the recombination occurring at the *pTET-lys2-oligo* allele with the insertion of 25 nt sequence (GATCTGTCCCTTACTAGCTAGGTAG) (61). *LYS2* gene itself is 60% AT and with the short insertion we considered this allele to be AT rich (‘A/T’ in Figure 1D).

CPT treatment led to induction of 9– and 6-folds of recombination at the *pTET-lys2-G/C* or *–A/T* construct, respectively. The CPT treatment was repeated for all four constructs, *pTET-lys2-GTOP, GBTM, -G/C* and *-A/T* after adding doxycycline to repress transcription from the *pTET* promoter. The increase in recombination frequency compared to DMSO-treated cells were between 1 and 2 fold and they were all significantly lower than when transcription was fully active (see P-values in Figure 1D). The level of transcription or the G/C content of the *pTET-lys2* reporter had no effect on the overall cell survival after CPT treatment (Supplementary Figure S3). Overall, the level of recombination induced by the Top1cc accumulation in CPT treated cells was not dependent on the sequence content but significantly dependent on the level of transcription. The lack of sequence-specificity for the Top1cc-induced hyper-recombination ensuing from CPT treatment (Figure 1D) or Top1Y722A expression (Figure 1C) suggests that Top1cc formation is not relevant to either suppression or aggravation of G4-associated genome instability. Top1 can interact with the C-terminal domain of elongating RNA polymerase (62), which could explain the enhanced level of recombination ensuing from CPT-induced Top1cc formation when transcription is activated.

### *E. coli* Top1 can partially complement the loss of Top1

**Figure 2.**
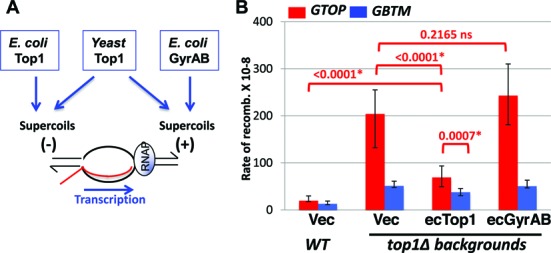
Expression of E. coli Top1 or Gyrases in *top1Δ* backgrounds. (**A**) Transcription-associated positive (+) and negative (–) helical stresses are removed by topoisomerases. (**B**) *WT* or *top1Δ* cells transformed with the indicated plasmid were grown in synthetic media lacking uracil supplemented with 2% galactose and 1% raffinose. Vec: YEP24. The rates of recombination at the *pTET-lys2-GTOP* (red bar) or *pTET-lys2-GBTM* (blue bar) cassette. The rates were determined by the method of median. Error bars indicate 95% confidence interval. P values (shown above red brackets) were determined by Mann-Whitney test using the Prism software. *, significantly different; ns, no significant difference.

During transcription, positive and negative supercoils accumulate ahead and behind of the RNA polymerase complexes, respectively (Figure 2A) (63). Because yeast Top1 can remove both negative and positive supercoils associated with transcription, it is not clear which type of supercoils contribute to the orientation-specific elevation in recombination for the *pTET-lys2-GTOP* construct in absence of Top1. We transformed *top1Δ* strains with plasmids expressing either *E. coli* Topoisomerase 1 (ecTop1), which removes only negative supercoils or a fusion protein of *E. coli* GyrA and GyrB (ecGyrase), which removes only positive supercoils (49,50). ecTop1 and ecGyrase were previously shown to be active in yeast and, furthermore, expression of either bacterial topoisomerase was shown to suppress the slow growth phenotype in strains lacking both yeast Top1 and Top2 activity. The recombination rates at *pTET-lys2-GTOP* or *-GBTM* expression did not significantly change upon expression of ecGyrase (Figure 2B). The expression of ecTop1 reduced the recombination rate at the *pTET-lys2-GTOP* by 3-fold compared to the control vector indicating that the removal of negative supercoils by ecTop1 can partially suppress elevated recombination at the highly transcribed G-run sequence in absence of yeast Top1.

### RNA:DNA hybrids or R-loops accumulate in an orientation-specific manner in *top1Δ* backgrounds

**Figure 3.**
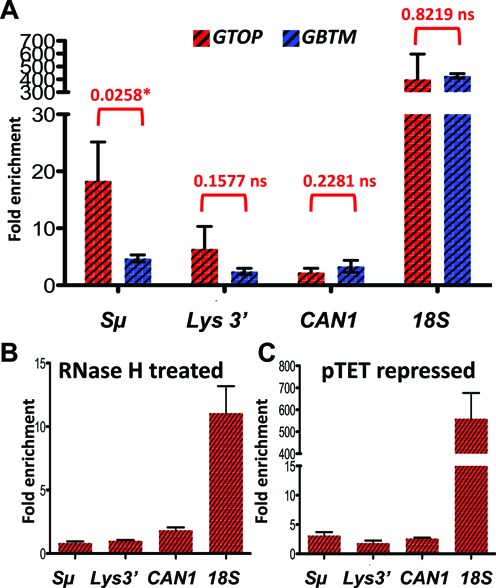
RNA:DNA hybrids in *top1Δ* backgrounds. RNA:DNA hybrids were quantitated by immunoprecipitation with S9.6 antibody as described in Materials and Methods. Fold enrichment relative to the untranscribed control region was determined by ΔΔCq analysis using the quantitative PCR results. Error bars indicate standard deviation calculated from three independent experiments. (**A**) Immunoprecipiation with samples from *top1Δ* cells containing either *pTET-lys2-GTOP* (red-hashed bars) or *pTET-lys2-GBTM* (blue-hashed bars). P values were determined by unpaired *t*-test using the Prism software. *, significantly different; ns, no significant difference. (**B**) DNA samples were treated with recombinant RNase H after sonication prior to immunoprecipitation. (**C**) Immunoprecipitation with S9.6 antibody was carried out in samples prepared from cells grown with 2 μg/ml doxycycline.

Annealing of the TS to the nascent RNA strand is one of the major consequences of the negative helical torsion accumulated behind the transcription complex in absence of Top1 activity. Accumulation of such extensive RNA:DNA hybrids or R-loops can lead to elevated genome instability (13,64). In order to determine whether there is a correlation between the observed increase in the recombination at the *pTET-lys2-GTOP* reporter and RNA:DNA hybrid accumulation, we used the genomic DNA isolated from *top1Δ* strains containing either the *pTET-lys2-GTOP* or *-GBTM* construct in immunoprecipitation (IP) experiment using the monoclonal antibody S9.6 followed by quantitative PCR (qPCR). S9.6 is specific for RNA:DNA hybrids of ≥6 bp in length (65,66) and was recently used in mapping RNA:DNA hybrid accumulation in the yeast genome (22,23). For the negative control, we carried out qPCR with primers targeting an untranscribed intergenic region on Chromosome V (23). At this region, ∼0.3% of DNA was recovered by the IP with S9.6 antibody. Due to the high G/C content and presence of repeated sequences, it was not possible to design efficient qPCR primers within the Sμ fragment in the *pTET-lys2-GTOP or -GBTM* constructs. Instead, a primer-pair targeting 100 nt upstream of 5′ end of the Sμ fragment was used to quantitate the RNA:DNA hybrids at the Sμ fragment (‘Sμ’ in the Figure 3A and Supplementary Table S1). When normalized to the negative control, there was a 18-fold enrichment of RNA:DNA hybrids proximal to the G-run containing Sμ fragment when it is in the context of the *pTET-lys2-GTOP*. When the Sμ fragment is in the *pTET-lys2-GBTM* context, the RNA:DNA hybrid proximal to the Sμ was enriched by ∼5-fold. We also quantitated the level of RNA by qRT-PCR methods using the identical primers used to detect RNA:DNA hybrids using two different housekeeping genes, *UBC6* and *ALG9*, as the reference controls (67) (Supplementary Table S2). No significant difference in the levels of RNA was present in the strains containing *pTET-lys2-GTOP* or *-GBTM* construct indicating that the preferential accumulation of RNA:DNA hybrids when the Sμ-fragment is in ‘GTOP’ configuration is not due to the difference in levels of transcription.

We additionally measured RNA:DNA hybrid accumulation within the *pTET-lys2-GTOP or-GBTM* construct but 3.5 kb downstream from the Sμ fragment (‘*LYS3*′’ in Figure 3A), at a RNA Polymerase II-transcribed gene with a low level of expression (‘*CAN1*’) and at the highly transcribed rDNA locus (‘*18S*’). Consistent with previously reported data, very high level of RNA:DNA hybrids were detected at the rDNA locus compared to the *CAN1* gene (Figure 3A). And at the three loci tested, there was no significant difference in the level of RNA:DNA hybrid accumulation between the strains containing the *pTET-lys2-GTOP* or *-GBTM* construct. R-loops are normally prevented by riboendonucleases called RNase H's that recognizes RNA:DNA hybrids and degrades the RNA strand. When the DNA samples were treated with recombinant bacterial RNase H prior to IP, RNA:DNA hybrids were detected at significantly reduced level at *Sμ* and at *18S* (*P* = 0.0111 and 0.0272, respectively) (Figure 3B). And when the cells cultured with doxycycline was used for IP with S9.6 antibody, RNA:DNA hybrids were reduced at the *Sμ* but not at the *18S* (*P* = 0.0164 and 0.9372, respectively) (Figure 3C).

### Recombination is elevated at G-run containing sequences in the absence of RNase H activity

**Figure 4.**
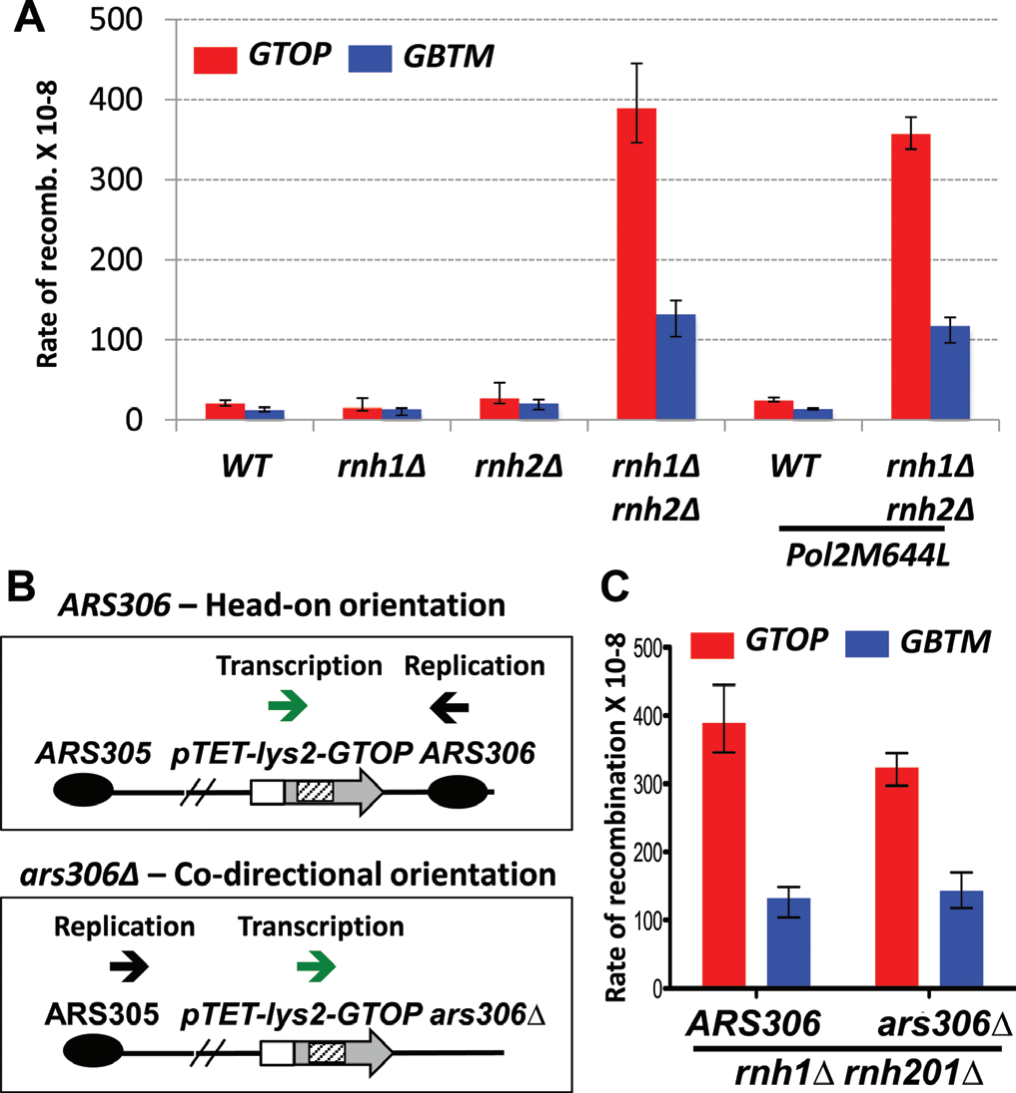
Effect of RNase H-disruption on the recombination rates. (**A**) The rates of recombination at the *pTET-lys2-GTOP* (red bar) or *-GBTM* (blue bar). Cells with indicated genetic backgrounds were grown in YEP supplemented with 2% glycerol and 2% ethanol. The rates were determined by the method of median. Error bars indicate 95% confidence interval. (**B**) Effect of changing the replication direction. The putative direction of transcription (green arrow) and replication (black arrow) through the *pTET-lys2-GTOP* or *-GBTM* construct are indicated for ARS306 or ars306Δ backgrounds. (**C**) The rates of recombination at the *pTET-lys2-GTOP* (red bar) or *-GBTM* (blue bar) in *rnh1Δ rnh201Δ ARS306* or *rnh1Δ rnh201Δ ars306Δ* backgrounds.

Similar to higher eukaryotic species, there are two RNase H class enzymes in yeast; RNase H1 is a single subunit protein encoded by the *RNH1* gene whereas RNase H2 is a hetero-trimer comprising gene products of the *RNH201, RNH202* and *RNH203* (68). RNA:DNA hybrids ≥3 bp-long including co-transcriptionally formed R-loops are substrates for both RNase H1 and H2 whereas single or double ribonucleotide(s) embedded in duplex DNA molecule is recognized by RNase H2 enzyme only (69). Single ribonucleotide-accumulation in DNA in absence of RNase H2 has been previously described as a key source of mutagenesis, recombination and gross chromosomal rearrangements in yeast (70–73) and the lesion responsible for the genome-wide chromosomal anomalies and embryonic lethality in mice (74). We previously showed that simultaneous disruption of RNase H1 and H2 results in significant elevation of recombination for both *pTET-lys2-GTOP and -GBTM* constructs (44). In order to determine whether the instability observed at these constructs correlates with the failure to remove co-transcriptionally formed R-loops or single ribonucleotides in DNA, we measured the effect of deleting *RNH1* or *RNH201* singly on the rate of recombination. In the single deletion mutants, *rnh1Δ* and *rnh201Δ*, there was no significant elevation in recombination for the *pTET-lys2-GTOP or -GBTM* construct (Figure 4A). As reported earlier, in strains deficient in both RNase H enzymes, the recombination rate for *pTET-lys2-GTOP* and *-GBTM* constructs were elevated by 19- and 11-fold, respectively (44). The functional redundancy of RNase H1 and H2 suggests that the elevated recombination is attributable to R-loops, the substrate common to both enzymes.

Single ribonucleotides embedded in DNA occur as a byproduct of DNA replication process. We introduced a single missense mutation (M644L) to the one of the two major replicative DNA polymerases Pol ϵ. Pol2M644L mutant was previously shown to have decreased propensity to incorporate ribonucleotides into DNA during replication and to reduce ribonucleotide-initiated mutagenesis (48,59). In *WT* or *rnh1Δ rnh201Δ* backgrounds, the recombination rates at the *pTET-lys2-GTOP or -GBTM* construct was not changed when Pol2M644L mutant was introduced (Figure 4A), further indicating that ribonucleotide incorporated in DNA is not a significant factor leading to the observed hyperrecombination at the highly transcribed Sμ fragment in *rnh1Δ rnh201Δ* background.

We also tested whether the relative orientation of transcription and replication affects the hyperrecombination observed at the *pTET-lys2-GTOP and –GBTM* constructs in RNase H-deficient yeast strains. Upon deletion of the replication origin *ARS306*, the genomic locus containing the *pTET-lys2-GTOP* or *-GBTM* construct is replicated from the *ARS305*, thereby reversing the direction of replication fork to move in co-directional orientation with the transcription initiating at the *pTET* promoter (Figure 4B). In *top1Δ* background, reversing the relative orientation of the transcription and replication from head-on to co-directional resulted in 3-fold decrease in the rate of recombination occurring at the *pTET-lys2-GTOP* reporter (45). In *rnh1Δ rnh201Δ* backgrounds, however, reversing the direction of the replication fork movement did not significantly change the recombination rate for the *pTET-lys2-GTOP* or *-GBTM* construct (Figure 4C).

### In absence of RNase H1 and H2, more extensive R-loop accumulation occurs when G-runs are on the NTS

**Figure 5.**
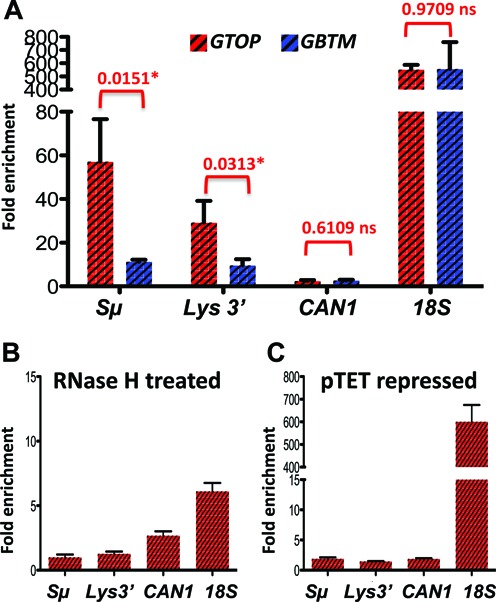
RNA:DNA hybrids in rnh1Δ rnh201Δ backgrounds. RNA:DNA hybrids were quantitated by immunoprecipitation with S9.6 antibody as described in the materials and methods. Fold enrichment relative to the untranscribed control region was determined by ΔΔCq analysis using the quantitative PCR results. Error bars indicate standard deviation calculated from three independent experiments. (**A**) Immunoprecipiation with samples from *rnh1Δ rnh201Δ* cells containing either *pTET-lys2-GTOP* (red hashed bar) or *pTET-lys2-GBTM* (blue hashed bar). *P* values were determined by unpaired *t*-test using the Prism software. *, significantly different; ns, no significant difference. (**B**) DNA samples were treated with recombinant RNase H after sonication prior to immunoprecipitation. (**C**) Immunoprecipitation with S9.6 antibody was carried out in samples prepared from cells grown with 2 μg/ml doxycycline.

In order to confirm that R-loop accumulation occurs as a consequence of deleting *RNH1* and *RNH201*, we quantitated the RNA:DNA hybrid that can be immunoprecipitated by the specific antibody S9.6. In the *rnh1Δ rnh201Δ* strains containing the *pTET-lys2-GTOP* or *-GBTM* construct, we detected greater than 500-fold enrichment of RNA:DNA hybrids at *18S*, compared to the untranscribed control region (Figure 5A). Proximal to the G4-forming Sμ within the *pTET-lys2-GTOP* and *-GBTM* constructs (*Sμ*), RNA:DNA hybrid was enriched by 57- and 11-fold compared to the negative control, respectively. At this site, the level of transcripts for the *pTET-lys2-GTOP* and *-GBTM* were indistinguishable to each other and to the level measured in *top1Δ* backgrounds (Supplementary Table S2). 3.5 kb away from the Sμ (*LYS3*′), the level of transcription was slightly higher in the strain containing the *pTET-lys2-GTOP* compared to that containing the *pTET-lys2-GBTM* construct, which could account for the significant difference in the level of RNA:DNA hybrid enrichment at the *LYS3*′ location (Figure 5A). As in *top1Δ* backgrounds, RNA:DNA hybrids at *Sμ* were significantly reduced by pretreating with recombinant RNase H or by repressing the *pTET*-driven transcription (*P* = 0.0075 and 0.0079, respectively) (Figure 5B and C).

### Removal of negative supercoils partly suppresses the elevated recombination at the G-run containing sequence in *rnh1Δ rnh201Δ*

**Figure 6.**
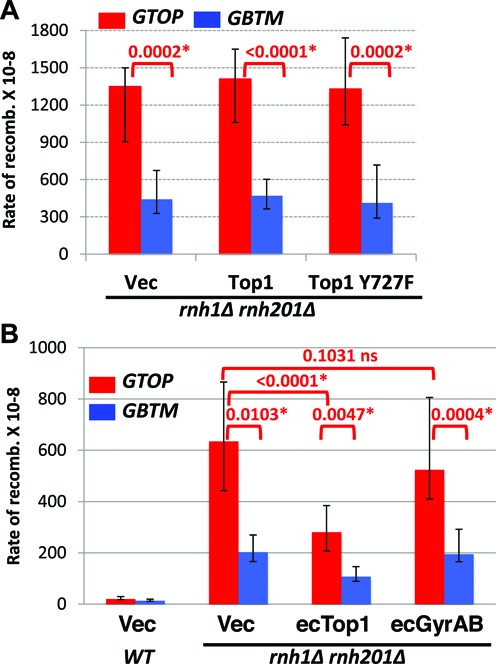
The rates of recombination in *rnh1Δ rnh201Δ* backgrounds. *WT* or *rnh1Δ rnh201Δ* cells transformed with the indicated plasmid were grown in synthetic media lacking uracil supplemented with 2% glucose (A) or 2% galactose and 1% raffinose (B). Vec: pRS416 for A; YEP24 for B. The rates of recombination at the *pTET-lys2-GTOP* (red bar) or *pTET-lys2-GBTM* (blue bar) cassette. The rates were determined by the method of median. Error bars indicate 95% confidence interval. *P* values (shown above red brackets) were determined by Mann–Whitney test using the Prism software. *, significantly different; ns, no significant difference.

As we reported earlier, the hyperrecombination at the *pTET-lys2-GTOP* and *-GBTM* constructs in *rnh1Δ rnh201Δ* backgrounds was suppressed by overexpression of yeast *RNH1* (45). In order to test whether reducing helical torsion in the highly transcribed region can counteract the defect in RNA:DNA hybrid processing in absence of RNase H activity, we transformed the plasmids expressing either yeast topoisomerase I or bacterial topoisomerases into *rnh1Δ rnh201Δ* cells. Expression of yeast Top1 (WT or Top1Y727F) or overexpression of the ecGyrase did not change the rate of recombination for either the *pTET-lys2-GTOP* or *-GBTM* construct (Figure 6A and B). Upon overexpression of ecTop1, which removes negative supercoils, the recombination rates for both the *pTET-lys2-GTOP* and *-GBTM* were reduced by ∼2-fold but remained significantly higher than the rates in *WT* backgrounds.

## DISCUSSION

When a repetitive sequence is actively transcribed, the formation of R-loops and DNA secondary structures could be mutually cooperative. Annealing of the nascent RNA to the TS, which initiates R-loop formation, might be promoted by the unavailability of the NTS when occupied in secondary DNA structure formation. Intra-strand interaction of the NTS, which is required for non-B structure formation, conversely, might be promoted by the unavailability of the TS when stably annealed to the RNA. For the G4 DNA-forming Ig switch region sequence, destabilizing the RNA:DNA hybrids involving the TS led to less robust G4 formation on the NTS (26). We previously found that disruption of RNase Hs or Top1, each of which was shown to lead to R-loop formation at highly transcribed areas such as the rDNA locus (18,22,23), elevates genome instability at a highly transcribed G4 DNA-forming Ig Sμ sequence in orientation-specific manner (44,45). R-loop and G4 DNA, which are likely to cooperatively form during active transcription of guanine-run containing sequence such as Sμ used to construct *pTET-lys2-GTOP* and *-GBTM* reporters, are each sufficient to induce genome instability (19,38,39). It is, therefore, difficult to define which of the higher order structures is directly responsible for the elevated recombination in *top1Δ* and/or *rnh1Δ rnh201Δ* backgrounds. We compared what factors affect the elevated recombination in *top1Δ* or *rnh1Δ rnh201Δ* strains in order to determine whether the intermediates involved in leading to instability at the G-run containing sequence in these two genetic backgrounds are identical or divergent.

We first confirmed whether robust transcription of the *pTET-lys2-GTOP* or *-GBTM* reporter results in the enrichment of RNA:DNA hybrids detectable by the monoclonal antibody S9.6. Our data show that, in both RNase H-defective and Top1-defective strain backgrounds, RNA:DNA hybrids accumulate to a significant extent proximal to the G/C rich Sμ insert within the *pTET-lys2-GTOP* where the transcription is oriented to produce (rG)n:(dC)n base pairing (Figures 3A and 5A). When transcription from the *pTET* promoter was repressed, no significant enrichment of RNA:DNA hybrids occurred relative to the untranscribed region on Chromosome V used as the negative control (Figures 3C and 5C). While in the *pTET-lys2-GBTM* context, the RNA:DNA hybrids accumulation proximal to the *Sμ* insert was observed at a much lower level in *top1Δ* and *rnh1Δ rnh201Δ* (Figures 3A and 5A). At the 3′ end of the *pTET-lys2-GTOP* reporter (*LYS3*′), where sequence is ∼60% A/T, RNA:DNA hybrids accumulated to much less degree than at *Sμ* in *top1Δ* and *rnh1Δ rnh201Δ* strains. In both strain backgrounds, the levels of steady-state transcripts were apparently about two-fold higher at *LYS3*′ than at *Sμ* (Supplementary Table S2) indicating that the level of guanine-content is the overriding factor in formation of R-loops. The orientation-bias or G-C bias in RNA:DNA hybrid accumulation was previously observed during *in vitro* transcription experiments and is consistent with the biochemical data showing that rG:dC pairing is thermodynamically more stable than rC:dG (26,54,75).

For the *pTET-lys2-GBTM* reporter in *rnh1Δ rnh201Δ* background, similar levels of RNA:DNA hybrid enrichment were observed at the *Sμ*, where the nascent RNA contains runs of Cs, and at the *LYS3*′, where the nascent RNA and TS strand are both A/T-rich (Figure 5A). In *top1Δ* cells, the level of RNA:DNA hybrids detected at *Sμ* and at *LYS3*′ within the *pTET-lys2-GBTM* construct was minimal and not significantly different from the level at the *CAN1* gene, which is transcribed at 20- to 30-fold lower rate than the *pTET-*derived reporter constructs (Figure 3A) (76). These results suggest that high number of Cs present on the NTS at *Sμ* within the *pTET-lys2-GBTM* is not sufficient to stimulate R-loop formation even when the rate of transcription is very high.

In correlation with the orientation bias in the recombination, R-loop accumulation in *top1Δ* and *rnh1Δ rnh201Δ* strains is equally pronounced when the transcription is oriented to place the guanine runs on the NTS (*GTOP*). However, our data indicate that there are substantial differences in the intermediate steps leading to elevated recombination in these genetic backgrounds. Unlike the disruption of RNase Hs, which elevates recombination of highly transcribed sequences containing biased runs of Gs or Cs or a mix of Gs and Cs (Figure 4 and (44)), the effect of Top1 disruption is highly specific to the Sμ fragment when the guanine-runs are located on the NTS. Several independent reports suggest that, for the most of the genome with the exception of the rDNA and *CUP1* loci, which contain arrays of direct repeats, the function of Top1 in maintaining genome stability is nonessential (58,77). The loss of Top1 did not result in significant elevation in recombination between two direct repeats (72), genome wide mitotic crossovers (58) or gross chromosomal rearrangements (GCRs) (73). But, as we have shown previously and in the current report, at the G-run containing sequence cloned from murine Ig Switch Mu region (Sμ fragment) or from the human protooncogene *TCF3*, Top1-disruption led to significant stimulation of gene conversion recombination and/or GCR (36,45). This indicates that the function of Top1 is specifically required to suppress genome instability when the sequences containing guanine runs are located on the NTS and therefore in conditions conducive to form G4 DNA structure during active transcription. Another important difference from *rnh1Δ rnh201Δ* is the significance of the R-loop in *top1Δ* background. According to previous experiments in yeast and mammalian cells, following the disturbance of a co-transcriptional process such as mRNP packaging, RNA-DNA unwinding, splicing, RNA degradation and export, the genome instability associated with R-loop accumulation was suppressed by overexpression of RNase H1 (15,19–21). RNase H1 overexpression was not able to affect recombination when it is elevated due to other factors such as defect in DNA damage response or repair pathways (21). We previously reported that preventing RNA:DNA hybrid accumulation by overexpression of *RNH1*, which significantly reduced recombination at both the *pTET-lys2-GTOP* and *-GBTM* constructs in *rnh1Δ rnh201Δ* background, failed to complement for the loss of Top1 in suppressing the elevated recombination at the *pTET-lys2-GTOP* construct (45).

In the absence Top1, R-loop accumulation is thought to occur as a secondary effect of the failure to remove negative supercoils accumulated during transcription. Negatively wound NTS limits the re-annealing with the TS thereby increasing the chance of TS annealing to the nascent RNA to form RNA:DNA hybrid behind the transcription complex. Negative superhelicity also leads to local melting of the duplex DNA and thereby facilitate the transformation of B-DNA into secondary structures including G4 DNA (78,79). We showed that removal of negative supercoils by bacterial topoisomerase I (ecTop1) can functionally compensate for the loss of yeast Top1 and results in reduction in the recombination rate at the *pTET-lys2-GTOP* reporter (Figure 2). Yeast Top1 expression from a plasmid resulted in complete complementation of *TOP1* deletion leading to the recombination rate indistinguishable from that in WT cells at the *pTET-lys2-GTOP* reporter (Figure 1B). With the ecTop1 expression, however, the recombination rate at the *pTET-lys2-GTOP*, although significantly less than in *top1Δ* cells, still remained ∼3.5-fold higher than WT level (Figure 2B). The inability of ecTop1 to fully complement the loss of yeast Top1 indicates that negative supercoil accumulation and the associated R-loop formation is only partially responsible for the genome instability associated with the highly expressed G-run containing sequence and that yeast Top1 has an as-yet-unknown function in addition to the removal of negative helical torsion. Alternatively, the partial complementation by ecTop1 and the complete lack of complementation by ecGyrase could be due to their limited activities in the particular yeast strain used in the current study, although the expression and function of the ecTop1 and ecGyrase in the heterologous yeast cells have been previously verified (49,50).

The effect of catalytically inactive Top1 (Top1Y727F) also suggests that Top1 function at the G-run containing sequence is not limited to removing helical torsion in DNA. When Top1Y727F was expressed in *top1Δ* cells with the *pTET-lys2-GTOP* reporter, there was 20- and 4.5-fold increase in recombination rate compared to *WT* and *top1Δ*, respectively (Figure 1B). When co-transcriptional formation of G4 DNA is much less likely because the guanine runs were located on the transcribed strand (*pTET-lys2-GBTM*), there was no significant difference in the rates of recombination in cells expressing no Top1 (*top1Δ* with the vector plasmid), WT Top1 (WT with the vector plasmid or *top1Δ* with the WT Top1-expression plasmid) or Top1Y727F (*top1Δ* with the Top1Y727F-expression plasmid). Since Top1Y727F failed to suppress genome instability at the highly transcribed Sμ sequence, it is clear that the catalytic activity of removing helical torsion is required for this function of Top1. The further increase observed in the rate of recombination at the *pTET-lys2-GTOP* with Top1Y727F expression in *top1Δ* backgrounds suggests that Top1Y727F mutant has an additional effect besides the lack of its catalytic properties. We speculate from this result that the role of Top1 could be more complex than its catalytic activity and involve its high-affinity, high-specificity binding of G4 DNA structures as demonstrated by the biochemical analyses with purified human or bovine topoisomerase I (80–82). G4 DNA-binding property of the mammalian topoisomerases I is expected to be shared by the highly conserved yeast Top1. The core and the catalytic domains of the human topoisomerase I, which together form a clamp around the duplex DNA, are 73% and 85% homologous to, respectively, and can functionally replace the corresponding yeast Top1 domains in chimeric protein constructs (Supplementary Figure S4)(83). In addition, out of the 26 residues directly contacting DNA according to the co-crystal structure of human Top1/DNA complex (42), 23 residues are identical in yeast Top1. In support of the direct interaction between Top1 and G4 DNA, chromatin immunoprecipitation (ChIP) analysis showed that both WT Top1 and Top1Y727F are enriched at the G-rich telomeres in yeast cells (84). Specific binding to G4 structure by WT Top1 could be required for recruitment of other factors that facilitate resolution of the secondary structure such as structure-specific helicases. When catalytically active, the dynamic nature of Top1–DNA interaction makes it possible that Top1 deposits and subsequently allows access of G4-resolving factors to the G4 DNA. The catalytically inactive Top1Y727F could exacerbate genome instability at G-run containing sequences by stabilizing the G4 DNA structures through cooperative binding and/or by preventing access of G4 processing factors such as structure-specific helicases. In addition, Top1Y727F, which forms an encircling clamp when bound to the canonical duplex DNA, could assume a different conformation when bound to the four-stranded G4 DNA (41).

In summary, using reporter constructs containing the identical G/C rich sequence from mouse Ig Sμ placed in two different orientations, we demonstrated the preferential accumulation of RNA:DNA hybrids when guanine-runs are located on the NTS of highly transcribed region. This specificity of RNA:DNA hybrid accumulation dependent on the transcriptional orientation correlates with the orientation-specific elevation in genome instability in absence of Top1 or RNase Hs. In *top1Δ* backgrounds, however, removal of negative helical torsion by overexpression of *E. coli Top1* and prevention of RNA:DNA hybrids by overexpression of RNase H1 had partial to no effect on the recombination occurring at the highly transcribed G4-forming sequence. This data suggest that accumulation of R-loops due to negative supercoils does not constitute the sufficient basis for the G4-specific genome instability in absence of Top1. Our current model is that yeast Top1 suppresses genome instability associated with guanine-runs first by preventing the G-runs present on the NTS from folding into G4 DNA through removal of the transcription-associated negative supercoils and also by recruiting proteins that can resolve G4 structures through physical interaction with G4 DNA. In the future, the technically challenging demonstration of the presence of G4 DNA structure and its physical interaction with Top1 and Top1Y727F *in vivo* will be necessary to provide concrete evidence for this model.

## Supplementary Material

SUPPLEMENTARY DATA
