# Supplementary material for: The role of topoisomerase I in suppressing genome instability associated with a highly transcribed guanine-rich sequence is not restricted to preventing RNA:DNA hybrid accumulation
Source: Nucleic Acids Res. 2015 Nov 2;44(2):718–29. doi: 10.1093/nar/gkv1152 (PMC4737143; doi:10.1093/nar/gkv1152)
Supplement: SUPPLEMENTARY DATA [file supp_44_2_718__index.html]

The role of topoisomerase I in suppressing genome instability associated with a highly transcribed guanine-rich sequence is not restricted to preventing RNA:DNA hybrid accumulation — SUPPLEMENTARY DATA 

# The role of topoisomerase I in suppressing genome instability associated with a highly transcribed guanine-rich sequence is not restricted to preventing RNA:DNA hybrid accumulation

## SUPPLEMENTARY DATA

- SUPPLEMENTARY DATA
