# Supplementary material for: The role of topoisomerase I in suppressing genome instability associated with a highly transcribed guanine-rich sequence is not restricted to preventing RNA:DNA hybrid accumulation
Source: Nucleic Acids Res. 2015 Nov 2;44(2):718–29. doi: 10.1093/nar/gkv1152 (PMC4737143; doi:10.1093/nar/gkv1152)
Supplement: SUPPLEMENTARY DATA [file supp_gkv1152_nar-01679-d-2015-File003.pdf]

Table S1. Primers used for qPCR and qRT-PCR

Name	Sequence	Target region
INTEG F*	GGCTGTCAGAATATGGGGCCGTAGTA	Intergenic region on chromosome V
INTEG R*	CACCCCGAAGCTGCTTTCACAATAC	
18S2-F**	TCCAATTGTTCCTCGTTAAG	"18S" 5' Region
18S2-R**	ATTCAGGGAGGTAGTGACAA	
LYS2 BGL F	GAGTAACCGGTGACGATGATATT	"Sμ" (5' of LYS2 gene; 100 nt upstream of Sμ insertion site)
LYS2 BGL R	CATTAAATGACCACGTTGGTTGA	
LYS2 STP F	GGACAGGTGTTGATTGGTCTAA	"LYS3'" (3' of LYS2 gene; 3.5 Kb away from Sμ)
LYS2 STP R	TCATTATGAGTTGGTGGAGGTAAA	
CAN1 F	GAGTTCTGGGTCGCTTCCAT	"CAN1" gene (YEL063C)
CAN1 R	GGCACCTGGGTTTCTCCAAT	
UBC6 F***	GATACTTGGAATCCTGGCTGGTCTGTCTC	UBC6 gene (YER100W)
UBC6 R***	AAAGGGTCTTCTGTTTCATCACCTGTATTTGC	
ALG9 F***	CACGGATAGTGGCTTTGGTGAACAATTAC	ALG9 gene (YNL219C)
ALG9 R***	TATGATTATCTGGCAGCAGGAAAGAACTTGGG	

Primers referenced from previous reports: *(1), ** (2), *** (3)

Table S2. The relative RNA levels of *lys2-GTOP* or *-GBTM* allele.

		Reference gene: *UBC6*		Reference gene: *ALG9*	
lys2 allele	Genetic Background	% Relative RNA* - Near Sµ (STD)**	% Relative RNA* - at LYS3' end (STD)**	% Relative RNA* - Near Sµ (STD)**	% Relative RNA* - at LYS3' end (STD)**
pTET-lys2-GTOP	*WT*	100 (±9.8)	100[#] (N.D.)	100 (±16)	100[#] (N.D.)
pTET-lys2-GBTM	*WT*	107 (±12)	75 (±24)	122 (±13)	94 (±40)
pTET-lys2-GTOP	*top1Δ*	92 (±31)	153 (±34)	101 (±14)	187 (±43)
pTET-lys2-GBTM	*top1Δ*	88 (±14)	53 (±8.8)	104 (±18)	68 (±12)
pTET-lys2-GTOP	*rnh1Δ rnh201Δ*	91 (±3.5)	165[#] (N.D.)	105 (±6.2)	196[#] (N.D.)
pTET-lys2-GBTM	*rnh1Δ rnh201Δ*	136 (±44)	93 (±29)	151 (±35)	110 (±24)

RNA was extracted using the standard hot acidic phenol method and treated with DNase I (New England Biolabs). Relative RNA levels were determined by quantitiative RT-PCR and ΔΔCq analysis using *UBC6* or *ALG9* as the reference gene. qRT-PCR was carried out using SensiFAST SYBR No-ROX One-Step kit from Bioline and Biorad CFX Connect instrument. RT-PCR conditions were as follows: 45°C for 10 mins, 95°C for 2 mins followed by 40 cycles of 95°C for 5 sec, 60°C for 10 sec, and 72°C for 5 sec. Primers for amplification of "Near Sµ" (LYS2 BGL F and LYS2 BGL R) are located ~ 250 NT from the 5' end of the *LYS2* mRNA and ~ 100 NT upstream of *GTOP* or *GBTM* cassette-insertion site. Primers for the amplification of "LYS 3'" (LYS2 STP F and LYS2 STP

R) are located ~4,000 NT from the 5' end of the *LYS2* mRNA. Primer sequences are listed in Table S1. *% was calculated by normalizing to the level of RNA in WT cells with *pTET-lys2-GTOP.*
**The standard deviations (STD) were calculated from three independent experiments except when indicated ([#]). [#]Two experiments were used to calculate mean expression level (standard deviation could not be determined – N.D.). When normalized to the transcription level at Sμ, the relative level of transcription at LYS3' in WT cells with *pTET-lys2-GTOP* was 156 ± 28% (*UBC6* as control) or 145 ± 7.7% (*ALG9* as control).

SUPPLEMENTAL FIGURE LEGENDS

Figure S1. Recombination Assay to test the effect of G-run containing sequence on genome stability in yeast.

The construction of plasmids and yeast strains involved in the designing of the recombination assay was previously described in detail (4).

A. Sequence of Sμ fragment. Sequence of the guanine-rich strand of the 770-bp fragment of mouse Ig Sμ region (Pubmed Accession # J00442) that was inserted into the *Bgl* II site of the *LYS2* gene is shown. In this orientation, the fragment comprises 137 A, 117 C, 358 G, and 158 T. Runs of ≥3 guanines are indicated in red.

B. Recombination Construct. The *lys2* allele with the insertion of Sμ fragment (hashed box) under the control of *pTET* promoter (*pTET-lys2-GTOP* or *–GBTM*) and the 3' truncated lys2 allele are located on the CHR III and CHR XV, respectively. Neither allele produces functional Lys2 protein (Lys^-). A Homologous recombination (HR) initiating at the *pTET-lys2-GTOP* or *–GBTM* construct and employing the truncated *lys2* allele on CHR XV as the donor sequence can be resolved either in crossover or gene conversion (non-crossover). Due to the orientations of the *lys2* alleles relative to the respective centromeres, crossover products of HR result in dicentric chromosome leading to inviable cells. Sμ insert is lost in the gene conversion product leading to the functional *LYS2* gene and the selectable Lys^+ phenotype.

Figure S2. Effects of Top1Y727F on the recombination rates under low transcription conditions or in WT strain background.

A. The rates of recombination under low transcription conditions. Empty vector (Vec – pRS416) or Top1 Y727F expressing plasmid was transformed into *top1*Δ cells. Growth media was synthetic media lacking uracil supplemented with 2% glucose and 2 µg/ml doxycycline (+Dox).

B. The rates of recombination in WT background. The rates determined after growth in synthetic media lacking uracil supplemented with 2% glucose. Vec: pRS416. P values (shown above red brackets) were determined by Mann-Whitney test using the Prism software. * - significantly different; ns - no significant difference.

Figure S3. Survival of CPT-treated cells.

WT cells containing the indicated *pTET-lys2*-derived reporter construct were treated either with DMSO or CPT (see material and methods). Percent survival was calculated relative to the DMSO treated cultures. Error bars indicate standard deviation from 8 to 24 independent measurements. For low transcription conditions (black bar), 2 µg/ml doxycycline was added to the growth media.

Figure S4. Alignment of human and yeast Top1.

The sequences of human and yeast topoisomerases I are located on the top and bottom lines of the alignment, respectively (5). Residues contacting DNA (6) are indicated in red (conserved) and in yellow (not conserved). A. The core domain (hTop1 residues 215 to 634). B. The C-terminal catalytic domain (hTop1 residues 712 to 765).

REFERENCES FOR THE SUPPLEMENTAL MATERIALS

1. Chan, Y.A., Aristizabal, M.J., Lu, P.Y., Luo, Z., Hamza, A., Kobor, M.S., Stirling, P.C. and Hieter, P. (2014) Genome-wide profiling of yeast DNA:RNA hybrid prone sites with DRIP-chip. *PLoS Genet*, **10**, e1004288.
2. El Hage, A., Webb, S., Kerr, A. and Tollervey, D. (2014) Genome-Wide Distribution of RNA-DNA Hybrids Identifies RNase H Targets in tRNA Genes, Retrotransposons and Mitochondria. *PLoS Genet*, **10**, e1004716.
3. Teste, M.A., Duquenne, M., Francois, J.M. and Parrou, J.L. (2009) Validation of reference genes for quantitative expression analysis by real-time RT-PCR in *Saccharomyces cerevisiae*. *BMC molecular biology*, **10**, 99.
4. Kim, N. and Jinks-Robertson, S. (2011) Guanine repeat-containing sequences confer transcription-dependent instability in an orientation-specific manner in yeast. *DNA Repair (Amst)*.
5. Larkin, M.A., Blackshields, G., Brown, N.P., Chenna, R., McGettigan, P.A., McWilliam, H., Valentin, F., Wallace, I.M., Wilm, A., Lopez, R. *et al.* (2007) Clustal W and Clustal X version 2.0. *Bioinformatics*, **23**, 2947-2948.
6. Redinbo, M.R., Stewart, L., Kuhn, P., Champoux, J.J. and Hol, W.G. (1998) Crystal structures of human topoisomerase I in covalent and noncovalent complexes with DNA. *Science*, **279**, 1504-1513.

Figure S1

A

```
                                    GATC GAGCTGAGCT GAGCTGGGTG AGCTGAGCTG
AGCTGAGCTG GGTGAGCTGA GCTGAGCTGA GCTGAGCTGG GTGAGCTGAG CTGAGCTGAG
CTGAGCTGAG CTGAGCTGGG GTGAGCTGGG CTGAGCTGAG CTGAGCTGGG GTGAGCTGAG
CTGAGCTGAG CTGAGCTGGG GTGAGCTGGG CTGAGCTGGG GTGAGCTGGG CTGAGCTGAG
CTGGGGTGAG CTGAGCTGAG CTGAGCTGAG CTGGGGTGAG CTGAGCTGAG CTGAGCTGGG
GTGAGCTGAG CTGGGCTGAG CTGAGCTGGG GTGAGCTGAG CTGAGCTGAG CTGAGCTGGG
GTGAGCTGAG CTGAGCTGAG CTGGGGTGAG CTGAGCTGGG GTGAGCTGAG CTGAGCTGGG
GTGAGCTGAG CTGAGCTGAG CTGGGGTGAG CTGAGCTGGG GTGAGCTGAG CTGAGCTGGG
GTGAGCTGAG CTGAGCTGGG GTGAGCTGAG CTGAGCTGGG GTGAGCTGAG CTGAGCTGGG
GTGAGCTGAG CTAGGGTGAG CTGGGCTGGG TGAGCTGGAG TGAGCTGAGC TGAGGTGAAC
TGGGGTGAGC CGGATGTTTT GAGTTGAGCT GGGGTAAGAT GAGCTGAACT GGGGTAAGAT
GGGATGAGCT GTGGTGAGGG GAGCTGGATT GAACTGAGCT GTGTGAGCTG AGCTGGGGTC
AGCTGAGCAA GAGTGAGTAG AGCTGGCTGG CCAAGAACCA AGAATCAATT AGGCTAAGTG
AGCCAGATTG CGCTGGGATC
```

B

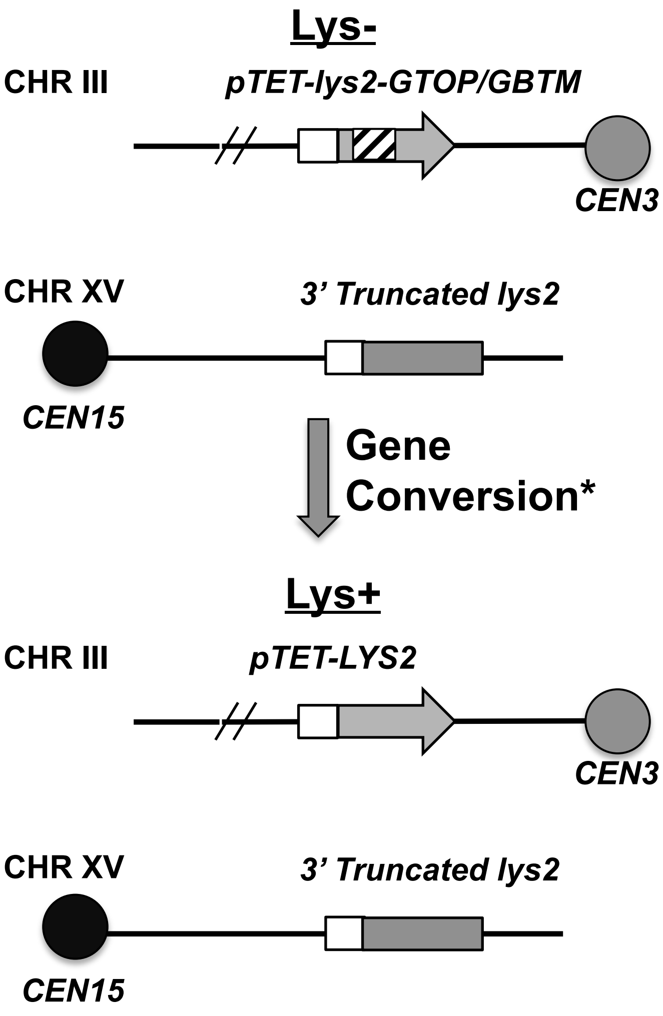

Figure S2

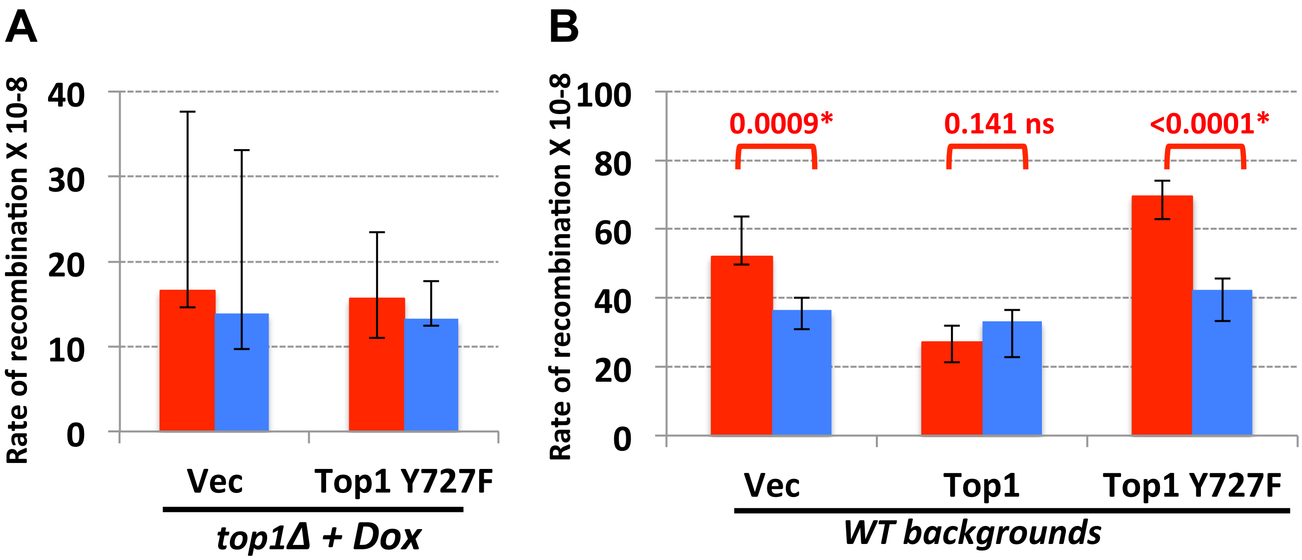

A
Rate of recombination X 10-8
40
30
20
10
0
Vec
Top1 Y727F
top1Δ + Dox
B
Rate of recombination X 10-8
100
80
60
40
20
0
0.0009*
0.141 ns
<0.0001*
Vec
Top1
Top1 Y727F
WT backgrounds

Figure S3

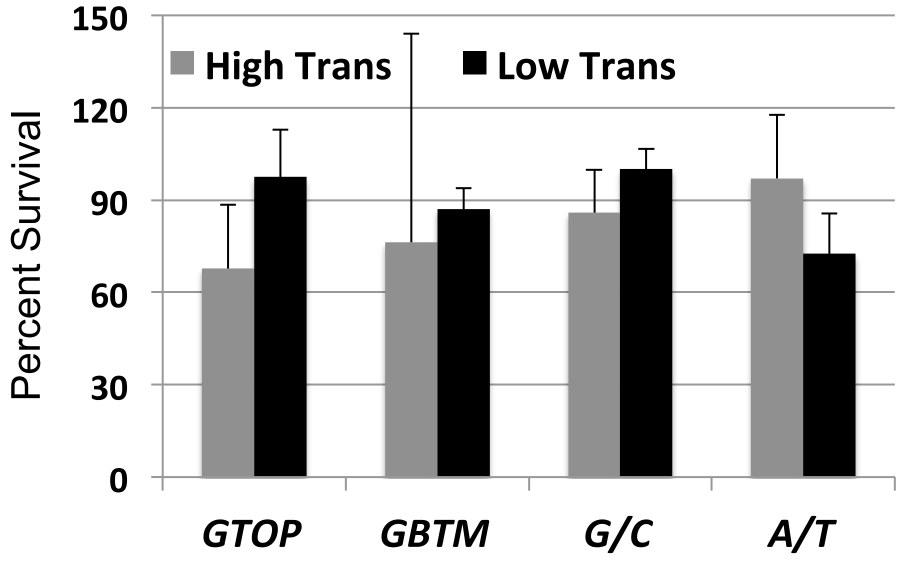
150
120
90
60
30
0
Percent Survival
High Trans
Low Trans
GTOP
GBTM
G/C
A/T

Figure S4

A. Core domain (53% identity; 73% homology)

```
IKWKFLEHKGPVFAPPYEPLPENVKFYYDGKVMKLSPKAEEVATFFAKMLDHEYTTKEIF
IKWVTLKHNGVIFPPPYQPLPSHIKLYYDGKPVDLPPQAEEVAGFFAALLESDHAKNPVF
***  *:*:* :*.***:***.::*:***** :.*.*:***** *** :*: :::.: :*

RKNFFKDWRKEMTNE----EKNIITNLSKCDFTQMSQYFKAQTEARKQMSKEEKLKIKEE
QKNFFNDFLQVLKESGGPLNGIEIKEFSRCDFTKMFDYFQLQKEQKKQLTSQEKKQIRLE
:****:*: : :.:.    :   *.::*:****:* :**: *.* :**::.:** :*: *

NEKLLKEYGFCIMDNHKERIANFKIEPPGLFRGRGNHPKMGMLKRRIMPEDIIINCSKDA
REKFEEDYKFCELDGRREQVGNFKVEPPDLFRGRGAHPKTGKLKRRVNPEDIVLNLSKDA
.**: ::* ** :*.::*::.***:***.****** *** * ****: ****::* ****

KVPSPPPGHKWKEVRHDNKVTWLVSWTENIQGSIKYIMLNPSSRIKGEKDWQKYETARRL
PVPPAPEGHKWGEIRHDNTVQWLAMWRENIFNSFKYVRLAANSSLKGQSDYKKFEKARQL
 **..* **** *:****.* **. * *** .*:**: * ..* :**:.*::*:*.**:*

KKCVDKIRNQYREDWKSKEMKVRQRAVALYFIDKLALRAGNEKEEGETADTVGCCSLRVE
KSYIDAIRRDYTRNLKSKVMLERQKAVAIYLIDVFALRAGGEKSE-DEADTVGCCSLRYE
*. :* **.:* .: *** *  **:***:*:** :*****.**.* : ********** *

HINLHPELDGQEYVVEFDFLGKDSIRYYNKVPVEKRVFKNLQLFMEN-KQPEDDLFDRLN
HVTLKPPN-----TVIFDFLGKDSIRFYQEVEVDKQVFKNLTIFKRPPKQPGHQLFDRLD
*:.*:*       .* **********:*::* *:*:***** :* .  *** .:*****:

TGILNKHLQDLMEGLTAKVFRTYNASITLQQQLKELTAPDENIPAKILSYNRANRAVAIL
PSILNKYLQNYMPGLTAKVFRTYNASKTMQDQLDLIPN-KGSVAEKILKYNAANRTVAIL
..****:**: * ************* *:*:**. :.  . .:. ***.** ***:****

CNHQRA
CNHQRT
*****
```

B. Cataytic domain (62% identity; 85% homology)

```
KQIALGTSKLNYLDPRITVAWCKKWGVPIEKIYNKTQREKFAWAIDMADEDYEF
SQVSLGTSKINYIDPRLSVVFCKKYDVPIEKIFTKTLREKFKWAIESVDENWRF
.*::*****:**:***::*.:***:.******:.** **** ***: .**::.*
```
